# Complex Seed Dormancy in *Parrotia subaequalis*: Identification, Breaking Mechanisms, and Conservation Strategies for an Endangered Species in China

**DOI:** 10.3390/plants14030452

**Published:** 2025-02-04

**Authors:** Yanfang Yang, Laikai Luo, Ling Zhu, Ying Cheng, Meng Yuan, Xiangdong Ruan, Kai Zhao

**Affiliations:** 1 Provincial Key Laboratory of Biodiversity Research and Ecological Protection in Southwest Anhui Province, College of Life Sciences, Anqing Normal University, Anqing 246133, China; yanfangyang@stu.aqnu.edu.cn (Y.Y.); laikailuo@stu.aqnu.edu.cn (L.L.); zhuling@stu.aqnu.edu.cn (L.Z.); chengying@stu.aqnu.edu.cn (Y.C.); yuanmeng@stu.aqnu.edu.cn (M.Y.); 2 The Belt and Road Model International Science and Technology Cooperation Base for Biodiversity Conservation and Utilization in Basins of Anhui Province, Anqing 246133, China; 3 Academy of Forestry Inventory and Planning, National Forestry and Grassland Administration of China, Beijing 100714, China

**Keywords:** *Parrotia subaequalis*, seed dormancy, germination, conservation

## Abstract

*Parrotia subaequalis*, an endangered plant unique to China, is highly valued for its significant ecological and ornamental value. The specific type of seed dormancy in this species has not been clearly reported, which limits its natural regeneration and artificial propagation, posing a challenge to its conservation and sustainable use. To address this, we conducted a comprehensive analysis of the seed dormancy mechanism of *P. subaequalis* and explored various methods to break dormancy, including cold and warm stratification, after-ripening, seed coat removal, and hormone soaking. Our analysis of the seeds’ physical properties, water absorption patterns, seed coat structure, embryo development, and endogenous inhibitors revealed that *P. subaequalis* seeds exhibit complex characteristics of physical and non-deep physiological dormancy. Experimental results showed that soaking the seeds in gibberellin (GA_3_) followed by seed coat removal effectively promoted germination. The optimal GA_3_ concentration for germination was 800 mg·L^−1^. Additionally, cold and warm stratification and after-ripening treatments significantly increased the germination percentage. These findings provide important technical support for dormancy release and seedling growth, which is crucial for the artificial propagation and population recovery of *P. subaequalis*.

## 1. Introduction

In the tapestry of global biodiversity, the conservation of endangered species is a thread that cannot be lost; once it is lost, the entire tapestry becomes fragile [1]. Among these species, *P. subaequalis* (Hamamelidaceae), a rare and endangered deciduous tree native to China, stands out [2]. As a relict angiosperm that survived the extinction of the dinosaurs 67 million years ago, this species is not only nationally protected but also occupies a unique position in evolutionary history [3,4]. Characterized by its precocious flowering before leafing, distinctive leopard-spotted bark, and large galls, *P. subaequalis* is an attractive candidate for ornamental plants and is a potential resource in various industries, possessing ecological and aesthetic value [5]. In recent years, due to human activities and other factors, this species has faced the dual threats of habitat loss and degradation, leading to a sharp decline in natural population numbers. It currently faces significant ecological challenges and urgently needs effective conservation efforts. Currently, asexual propagation techniques for *P. subaequalis* include cuttings and grafting, but large-scale seedling production still relies on seed propagation. Traditional seed propagation methods for *P. subaequalis* involve long-term stratification or layering treatments, but issues such as low germination rates, long germination periods, and uneven seedling emergence are common. These signs indicate that the seeds of *P. subaequalis* have certain dormancy characteristics, and the problem of seeds not germinating normally seriously constrains production and population recovery [6,7,8,9]. To conserve rare and endangered plants, merely collecting and storing seeds may not be sufficient, as seeds must ultimately germinate to support population recovery [10]. However, current research on the dormancy types and germination conditions of *P. subaequalis* seeds remains very limited, and these unknown aspects seriously hinder the development of effective dormancy-breaking strategies and the strengthening of populations.

The study of seed dormancy mechanisms is significant for understanding the ecological adaptation and evolutionary strategies of plants, as well as for formulating effective conservation measures. Considering the survival and recovery of *P. subaequalis* populations, as well as the health of the ecosystems they inhabit, it is necessary to emphasize the ability to understand and manipulate the complex mechanisms of seed dormancy and germination. The phenomenon of viable seeds (or other germination units) failing to germinate under favorable environmental conditions (such as temperature, light, etc.) is called seed dormancy [11,12,13,14]. As a complex ecological adaptation mechanism that has evolved in plants, this phenomenon plays a key role in ensuring the successful germination of seeds under favorable conditions and avoiding adverse environments [15,16]. By considering the permeability of the seed coat, the degree of embryo development, and the seed’s response to GA_3_ [17], after-ripening, and stratification treatments, the inducing factors and types of seed dormancy can be determined [12,18]. Seed dormancy is divided into several different types: physiological dormancy (PD), morphological dormancy (MD), morphophysiological dormancy (MPD), physical dormancy (PY), and combined dormancy (PY+PD) [11,12]. Research on the seed dormancy of *P. subaequalis*, a monogeneric and monotypic plant of significant phylogenetic importance, will greatly promote the study of seed dormancy evolution [5,19,20]. Moreover, *P. subaequalis* and *Parrotia persica* form an East Asia–West Asia disjunct distribution pattern. The seed dormancy type of *P. persica* has been identified as non-deep physiological dormancy (Type 3) [21]. Comparing the seed dormancy types of these two species helps to explore their evolutionary relationships from a seed biology perspective, providing new clues and evidence for the study of the origin, evolution, and geographical distribution of plants. At the same time, it can also help to better understand how both species adapt to environmental changes through seed dormancy in different ecological environments, increasing their chances of survival and reproduction, thereby providing deeper insights into plant ecological adaptation [22].

This study aims to fill the knowledge gap by conducting a comprehensive analysis of the dormancy characteristics of *P. subaequalis* seeds, combining existing research with a series of dormancy experiments to classify the dormancy categories of these seeds. Our goal is to identify the types of seed dormancy present in *P. subaequalis*, assess the effectiveness of various dormancy-breaking methods, and evaluate their impact on seedling development [15,21,23]. To achieve this, we examined several key aspects: (1) the physical properties of seeds that may affect dormancy, (2) the rate and pattern of water uptake by seeds, which are crucial for initiating metabolic processes, (3) the seed coat structural characteristics that may indicate morphological dormancy, (4) the morphological anatomy of seeds to understand the stages of embryo development, (5) the endogenous inhibitors present in seed extracts that may be responsible for physiological dormancy, and (6) the techniques that can be employed to induce dormancy release, such as warm and cold stratification, seed coat removal, gibberellin soaking, and after-ripening after dry storage.

## 2. Results

### 2.1. Seeds’ Physical Properties

**Figure 1:**
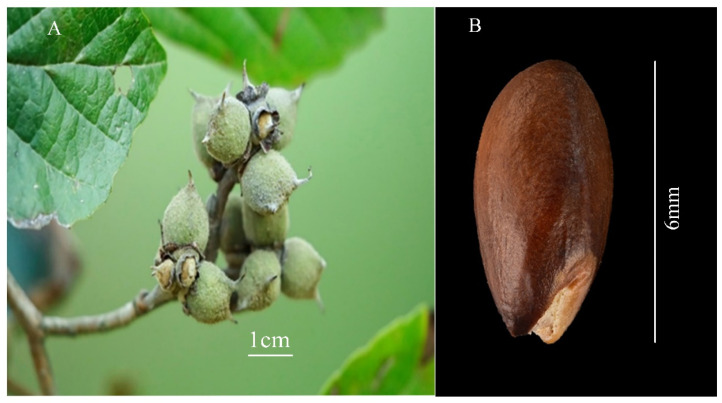
The left panel (**A**) illustrates a capsule fruit of *P. subaequalis*, featuring one chamber and two compartments, while the right panel (**B**) presents the mature seeds of the species.

As shown in Figure 1, the seeds of *P. subaequalis* are spindle-shaped, smooth in surface, and have a hard seed coat. The main color of the seeds is brown, except for the white area around the hilum. The average weight of 1000 seeds is 35.22 ± 0.24 g, indicating a low degree of variation in seed mass (Table 1). The length of the seeds is 7.40 mm, and the width is 3.17 ± 0.13 mm (Table 1), showing a relatively uniform seed size. The TTC staining results show a high seed viability of (95.3 ± 1.2)%, indicating good health of the seeds.

### 2.2. Seed Moisture Content and Water Absorption Curve

**Figure 2:**
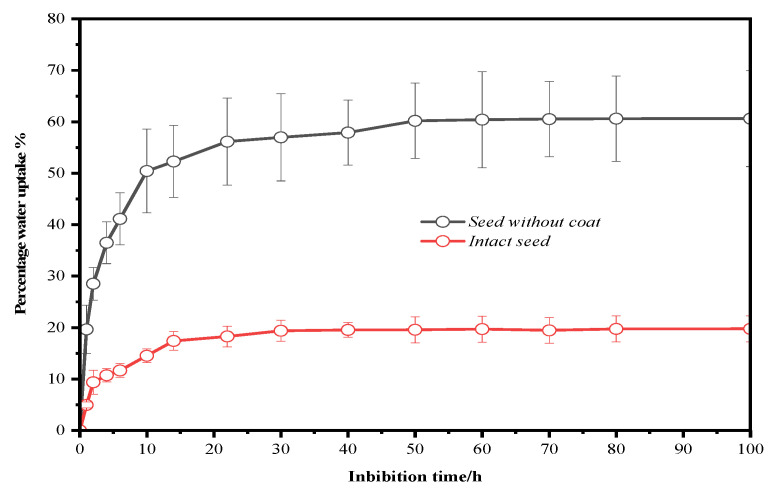
Changes in water absorption rate (mean ± S.D. for three replicates) of *P. subaequalis seeds*.

Measurements indicated that the initial moisture content of the seeds was 4.12 ± 0.3%. The water absorption curve, as shown in Figure 2, clearly shows three stages: a rapid water absorption period, a slow water absorption phase, and a stable phase. Both intact seeds and seeds with the seed coat removed were in the rapid water absorption phase for the first 10 h. After 10 h, they gradually transitioned from rapid to slow water absorption until 100 h of water absorption, at which point both treatments transitioned from slow to stable absorption. During the water absorption process, the percentage of water absorbed by the seeds with the seed coat removed was significantly higher than that of the intact seeds. This result indicates that although the seed coat of *P. subaequalis* seeds has some permeability to water, it significantly hinders the absorption of water by the embryo.

### 2.3. Seed Structure

**Figure 3:**
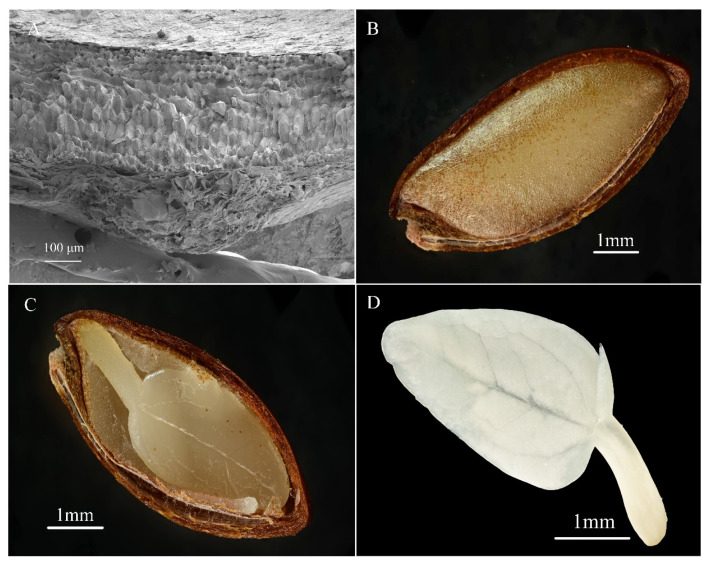
The group of figures shows the seed structure of *P. subaequalis*: (**A**) is a scanning electron microscope image of the seed’s outer seed coat, (**B**) shows the surface of the seed’s inner seed coat, (**C**) is a cross-section of the *P. subaequalis* seed, and (**D**) is the complete embryo of *P. subaequalis*.

From Figure 3A, ultrastructural observation of the *P. subaequalis* seed coat reveals that it is composed of four layers of cells, which can be distinctly divided into six parts: the outer surface of the seed coat, the cuticle, the compact layer of small stone cells, 3–5 layers of large stone cells, another compact layer of small stone cells, and the inner part of the seed coat. Overall, the seed coat of *P. subaequalis* has a high degree of lignification and a dense structure. These special multi-layered, tightly arranged, solidified structures can effectively control the permeability of water and air and protect the seeds from adverse environmental influences. As shown in Figure 3B, the hard seed coat encloses the internal endosperm, and on the surface of the endosperm, there is a thin film-like structure, which is the remnant of the nucellar tissue. Brown spots are attached to the film-like structure, which are formed by the adhesion of the seed coat to the film. The longitudinal section of the *P. subaequalis* seed shown in Figure 3C indicates that the freshly matured seeds have a fully developed embryo, which is wrapped by the endosperm in a spade-like shape (Figure 3D), and is morphologically classified as type FA1.

### 2.4. The Effect of Inhibitors on the Germination of Brassica rapa var. glabra Seeds

From Table 2, it can be seen that different concentrations of *P. subaequalis* seed coat extracts (T1: 25%, T2: 50%, T3: 75%, T4: 100%) have an inhibitory effect on the germination of *B. rapa* var. *glabra* seeds, especially at high concentrations (T4: 100%), where the germination rate is reduced to 21.4%. As the concentration of silver-thread plum seed coat extracts increases, the germination rate, germination potential, and germination index of *B. rapa* var. *glabra* seeds are all inhibited, and the inhibitory effect on the radicle of *B. rapa* var. *glabra* seedlings is more pronounced. This suggests that the extracts from the silver-thread plum seeds may contain strong inhibitors that can suppress the germination and growth of *B. rapa* var. *glabra* seeds and seedlings. It is speculated that silver-thread plum seeds may have an endogenous physiological dormancy mechanism, which may be related to the seed’s own physiological activities to protect the seeds from germinating under adverse conditions.

### 2.5. Seed Germination Under Various Treatments

Germination of seeds with seed coat removed: The germination test of manually dehulled *P. subaequalis* showed that the final germination rate was (42.7 ± 5.0)%, with a germination period of 20 days. The control group showed no germination within 30 days.

Germination of seeds after dry after-ripening treatment: As shown in Table 3, the dry after-ripening treatment has a significant promoting effect on the dormancy and germination of *P. subaequalis* seeds. Among them, the final germination percentage of the seeds after 90 days of dry after-ripening treatment was the highest, at (25.3 ± 1.2)%, which was higher than the control’s (17.3 ± 1.1)%. The germination rates of all dry after-ripening treatments that were less than 90 days were significantly higher than those of the control group. Even when the treatment period was extended to 120 days, the final germination percentage of *P. subaequalis* seeds was still slightly higher than that of the control group.

Cold and warm stratification treatments on the germination of *P. subaequalis* seedlings: According to Table 4, both cold and warm stratification can promote the germination of *P. subaequalis* seeds. Specifically, as the cold stratification treatment time increases from 30 days to 120 days, the germination percentage gradually increases from 18.0% ± 1.0% to 23.7% ± 3.1%. This indicates that under the low-temperature condition of 4 °C, extending the treatment time helps to improve the germination percentage of the seeds, but the increase is relatively small. At the same treatment time, the germination percentage of the 15 °C treatment is 2.7% to 5.0% higher than that of the 4 °C treatment. After 120 days of warm stratification treatment, the germination percentage reached the highest among all treatments, at 28.7% ± 3.1%.

**Figure 4:**
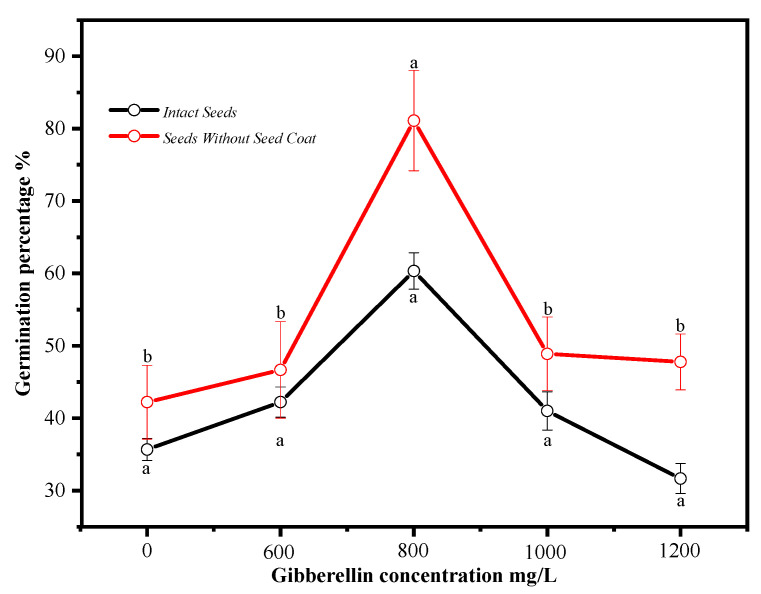
Germination percentage of *P. subaequalis* seeds treated with different concentrations of gibberellic acid. The superscript letters in the figure indicate significant differences among the values within the same column (*p* < 0.05).

As shown in Figure 4, the removal of the seed coat significantly enhanced the germination rate of *P. subaequalis* seeds, reaching 42.7% within a 20-day germination period, while the untreated control group showed no germination within 30 days. Furthermore, treatment with 800 mg·L^−1^ gibberellin (GA_3_) further improved the germination effect, increasing the germination rate to 81.1%.

## 3. Discussion

Under normal circumstances, we can determine the dormancy status and degree of dormancy of freshly matured seeds by conducting a 30-day germination test under optimal germination conditions. The types of dormancy and germination methods include in vitro embryo culture, seed coat water absorption curve measurement, seed coat scarification, stratification treatment, gibberellin (GA_3_) soaking, germination response after-ripening treatment, chemical treatment, and molecular biological treatment [12,15,16,24,25,26]. In this study, the viability of the *P. subaequalis* seeds was as high as (95.3 ± 1.2)%, with uniform size and plump characteristics. However, the germination rate was 0 in the 30-day germination test for seeds sown immediately after collection, indicating that *P. subaequalis* seeds have certain dormancy characteristics [27]. This is consistent with the speculation by Hu Guowei and other scholars [6]. The water absorption curve was measured, and the difference in water absorption between seeds with and without seed coats confirmed that the seed coat hinders seed water absorption [28]. Observations revealed that the seeds of *P. subaequalis* are dark in color, and the physical dormancy and overwintering ability of dark seeds may be related to the seed coat. Dark seed coats can give these seeds lower permeability, delaying and slowing germination. Further observations found that the seed coat of *P. subaequalis* has a multi-layered special solidified structure, indicating a high degree of lignification, which hinders the absorption of water by the embryo. It is speculated that the multi-layered, tightly lignified structure of the seed coat may be an adaptation of *P. subaequalis* to environmental selection pressures. This structure can effectively control the permeation of water and air, protecting the seeds from adverse environmental conditions. This is consistent with the concept of seed dormancy as an adaptive strategy for plants to cope with stress and unpredictable environments [29,30,31,32]. For seeds with physical dormancy, removing the seed coat can release the mechanical constraint and thus achieve germination [33]. In this study, the final germination rate of seeds with the seed coat removed within a 20-day germination period was (42.7 ± 5.0)%, while the control group showed no germination within 30 days, indicating that removing the seed coat can release physical dormancy and greatly shorten the germination period [33]. According to Baskin and Baskin’s seed dormancy classification system [12,24], freshly matured *P. subaequalis* seeds exhibit characteristics of physical dormancy.

Extracts from the seed coat of *P. subaequalis* significantly inhibited the germination of Chinese cabbage seeds, indicating the presence of endogenous inhibitors within the seed coat. We speculate that *P. subaequalis* has endogenous physiological dormancy [34]. Subsequent after-ripening treatment further confirmed that *P. subaequalis* seeds may have an endogenous physiological dormancy mechanism, which may be related to the physiological activities of the seeds themselves to protect the seeds from germinating under adverse conditions [15]. In addition, in the warm and cold stratification treatments, the germination of *P. subaequalis* seeds showed positive effects. The results of warm and cold stratification treatments indicate that higher temperatures (15 °C) are more conducive to seed germination than lower temperatures (4 °C), which may be because higher temperatures help the activity of enzymes within the seeds, thereby promoting the metabolic processes and germination of the seeds [35]. The annual average temperature in the growth area of *P. subaequalis* is usually between 10 °C and 15 °C. This temperature range is suitable for its slow growth and long-term survival. A stratification treatment of 120 days at 15 °C provided the best germination environment for the seeds, which may be because this combination of stratification temperature and time provided the seeds with the best physiological activation conditions and energy reserves [13,34].

According to the three definitions of physiological dormancy, non-deep simple physiological dormancy, morphophysiological dormancy, and deep simple physiological dormancy [36], we note that seeds with deep physiological dormancy do not respond positively to gibberellin (GA_3_), but seeds with non-deep and intermediate physiological dormancy are stimulated by GA_3_ [37]. In addition, after-ripening and stratification treatments are only referred to as dormancy-breaking treatments in seeds with non-deep physiological dormancy [23,34].

*P. persica* mainly grows in the Hyrcanian forests of northern Iran and the Talish Mountains of Azerbaijan, which are temperate forest types. This environment provides relatively stable temperature and humidity conditions, reducing the impact of extreme climates. The microclimate in the forest understory helps maintain soil moisture and reduce water evaporation. The above forest structure, climate conditions, and ecological niche selection are highly consistent with the current ecological adaptation environment of the endangered plant *P. subaequalis* in China. In terms of seed morphology, both have similar FA1-type embryos that are fully developed at maturity, and neither exhibits post-maturity embryo development, i.e., neither *P. subaequalis* nor *P. persica* has morphological dormancy [38].

Intriguingly, during our research, we observed that the branches of *P. subaequalis* often bear large galls, which are typically irregularly shaped and resemble papayas. This unique feature has led to the local nickname of “*Carica papaya*” for this species. However, there is currently no documented evidence regarding whether *P. persica* also develops such large galls. Upon further dissection of the galls on *P. subaequalis*, we discovered thousands of aphids inside. We hypothesize that these aphids feed or lay eggs on the trunks and larger branches of *P. subaequalis*, stimulating the accelerated division and abnormal differentiation of phloem cells, which subsequently leads to gall formation. Additionally, *P. subaequalis* has highly specific growth requirements, and its unique environment may also influence the parasitic behavior of insects, thereby promoting gall development. To date, we have not conducted an in-depth investigation into the impact of galls on seed germination. However, based on existing studies by both domestic and international scholars, we speculate that the germination of *P. subaequalis* seeds may be affected by gall formation in two primary ways: First, galls impact seed development by altering nutrient allocation and reducing seed quality; they also cause metabolic changes and deform plant organs, indirectly affecting seed formation. Second, galls may disrupt the balance of plant hormones, deepen seed dormancy, reduce seed vigor, and lead to decreased germination rates [25,39]. The specific mechanisms of these effects vary depending on the plant species, gall size and number, and environmental conditions. We plan to conduct further in-depth research on this topic in the future.

Finally, seed dormancy plays a crucial role in the adaptation and evolution of seed plants. While its biological significance is relatively well understood, the molecular mechanisms underlying the induction, maintenance, and alleviation of seed dormancy remain incompletely elucidated. In the future, we intend to employ omics technologies to further investigate the molecular mechanisms of dormancy alleviation and germination in the seeds of the rare and endangered plant *P. subaequalis*, with the goal of providing a scientific basis for the conservation and propagation of this species [40].

## 4. Materials and Methods

### 4.1. Sample Source and Acquisition

The seeds of *P. subaequalis* were collected in November 2022 from Huangwei Town, Yuexi County, Anqing City, Anhui Province (116°11′–116°22′ E, 30°51′–31°11′ N, altitude 300–700 m). Fresh mature seeds were air-dried indoors (at a temperature of 25 °C and a relative humidity of 50%) for two weeks before being used for subsequent experiments. Fresh seeds were subjected to germination experiments, and the remaining seeds were stored in a refrigerator at 4 °C and sealed in glass jars to reduce respiration and maintain seed viability. The TTC staining method was used to determine seed viability [41].

### 4.2. Morphological Index Measurement

The “hundred-seed mass method” was used to determine the weight of the seeds. One hundred seeds were randomly selected and their total weight was measured using an analytical balance. This measurement was repeated five times, and the average weight was calculated. The weight data of the hundred seeds were then multiplied by 10 to determine the thousand-seed mass of *P. subaequalis* seeds. The length and diameter of the seeds were measured using a vernier caliper. Thirty seeds were measured in each group, and this process was repeated three times to determine the average dimensions. Other physical characteristics of the seeds, such as color, luster, shape, and plumpness, were also observed and recorded.

### 4.3. Seed Moisture Content and Water Absorption Curve Determination

According to the drying weighing method recommended by the International Seed Testing Association (ISTA, Zurich, Switzerland, 2021), the initial moisture content of the seeds was calculated. To detect whether the seed coat and endosperm would cause a barrier to seed water absorption, the water absorption percentage change of intact seeds and seeds with the seed coat removed during the culture period was determined according to Li’s method [19], and the water absorption curve was plotted. Each experimental treatment was repeated three times, with ten seeds per repetition placed in a beaker containing 50 mL of distilled water and cultured in a temperature-controlled environment set at 25 °C. The seeds were taken out at certain intervals, surface moisture was absorbed with absorbent paper, and the seed mass was measured. The weighing intervals were adjusted according to the progress of the experiment until the seed mass no longer increased. The water absorption percentage of the seeds was calculated using the following formula: Water absorption percentage = (Weight after absorption − Initial weight)/Initial weight × 100%. The germination rate (GR) of the seeds was calculated using the following formula: Germination rate = (Number of germinated seeds/Total number of seeds tested) × 100%.

### 4.4. Seed Structure

Ultrastructural observation of the seed coat: Freshly collected *P. subaequalis* seeds that had been air-dried naturally for two weeks were manually peeled using a blade. These peeled seed coats were cut into 2 × 2 mm pieces, fixed with a 2.5% glutaraldehyde solution, and then gradient dehydrated in acetone. The samples were then freeze-dried at a temperature of −4 °C and gold-coated using an ion sputter coater. The ultrastructure of these samples was observed and photographed under a JSM-7800F field emission scanning electron microscope (JEOL Ltd., Tokyo, Japan) and a Phenom XL G2 desktop scanning electron microscope (SEM, Thermo Fisher Scientific, Waltham, MA, USA), revealing the complex details of the seed coat’s microstructure. Regarding observation of internal seed morphology, for the morphological assessment of the seeds, longitudinal sections were made and micro-stacking photography was used to capture the internal structure of the seeds.

### 4.5. Bioactivity Assay of Inhibitors

Newly harvested *P. subaequalis* seeds were selected and the seed coat was separated from the embryo, then weighed separately on an electronic analytical balance (with a precision of 0.0001 g). The seed coat was ground and placed in a beaker, ten times the mass of distilled water was added, and the mixture was extracted at room temperature, stirring regularly to ensure full extraction. Then, the extract was centrifuged at 8000 r/min for 3 min using a high-speed refrigerated centrifuge, and the supernatant was taken as the stock solution. The stock solution was diluted to concentrations such as 25%, 50%, or 75%, labeled, and sealed in reagent bottles for low-temperature storage. *B. rapa* var. *glabra* seeds were selected as the receptor material for bioactivity determination. A total of 2 mL of the *P. subaequalis* seed coat extract was evenly added to Petri dishes lined with two layers of filter paper fully moistened with distilled water. The same volume of distilled water was treated as a control, and sealed with a film to prevent water evaporation. Three replicates were set up for each treatment, with 50 plump *B. rapa* var. *glabra* seeds for each replicate, cultured at a constant temperature of 25 °C, and the final germination rate of the *B. rapa* var. *glabra* seeds was counted, totaling 10 days. Germination was defined as the radicle elongation ≥2 mm. The germination rate, germination potential, germination index, root length, and other indicators were calculated, where germination potential = (number of germinated seeds/total number of seeds) × 100%; Germination index = total days ∑(Gt ÷ Dt) (Gt is the number of seeds germinated each day within the final period of the germination test, Dt is the number of germination days, and ∑ is the sum).

### 4.6. Germination Test

#### 4.6.1. Seed Coat Removal

Seeds that were freshly matured and naturally air-dried for two weeks were treated by peeling the seed coat using a blade or other tools. After dehulling, the surface seed coat fragments were cleaned to maintain germination potential. The intact seeds were used as a control group for the germination experiment. The number of days to germinate and the germination rate were observed and recorded, with three replicates per group and 50 seeds per replicate.

#### 4.6.2. Dry After-Ripening Treatment

Freshly harvested mature seeds that had been naturally air-dried for two weeks were subjected to after-ripening treatment under conditions of 15 °C and 50% humidity. Samples were taken for germination tests after 30, 60, 90, and 120 days, respectively. Each treatment had three replicates, with 50 seeds per replicate. Seeds that were freshly harvested and air-dried for two weeks were directly sown as a control.

#### 4.6.3. Gibberellin Soaking Treatment

Seeds of uniform size were selected and disinfected with 75% alcohol for 5 min. They were rinsed with sterile water three times. Then, they were disinfected with 30% sodium hypochlorite for 30 min and rinsed with sterile water five times. After disinfection, the seeds were treated in GA3 solutions at concentrations of 600, 800, 1000, and 1200 mg·L^−1^ for 48 h. The soaking temperature was set at 36 °C. After soaking, the seeds were rinsed with distilled water three times to remove any residual hormones on the surface. For some seeds, after soaking, the seed coat was removed with a knife and germination tests were conducted. For others, the seeds were kept intact for further germination trials. Both types of seeds were germinated in Petri dishes at the optimal temperature (25 °C), humidity (75%), and light (4000 lx). The filter paper was kept moist when adding sterile water daily, and it was replaced promptly if mold appeared. For each treatment, the germination test was conducted in triplicate, with 30 seeds per replicate. The control for both materials was seeds soaked in distilled water for 48 h.

#### 4.6.4. Cold/Warm Stratification Treatment

Seeds that had been naturally air-dried for two weeks were cleaned and disinfected, then mixed with moist sand (with a water content of 70%) in a certain ratio (5–10 times) and thoroughly mixed. The mixed seeds and sand were placed into a black plastic box. They were then stored at 4 °C and 15 °C for cold stratification and room temperature stratification treatments, respectively. After stratification for 30, 60, 90, and 120 days, samples were taken and germinated at the optimal temperature of 25 °C. Each experimental treatment had three replicates, with 50 seeds per replicate. Both treatments used seeds that were freshly harvested and air-dried for two weeks as a control.

### 4.7. Data Processing and Analysis

Experimental data were organized using Excel 2021, and charts were created using Origin 2021. Statistical analysis was conducted using SPSS 26.0. The statistical analysis included performing a one-way ANOVA to determine significant differences, followed by post hoc multiple comparisons using the least significant difference method (LSD-Test).

## 5. Conclusions

In summary, *P. subaequalis* seeds exhibit complex dormancy characteristics, including physical dormancy and non-deep physiological dormancy. The most effective method to relieve dormancy is the combination of GA_3_ soaking and seed coat removal treatment. The findings of this study provide important technical support for the artificial propagation and population recovery of *P. subaequalis*, which is of great practical significance for the protection of this endangered species. Future research can further explore the molecular mechanisms of *P. subaequalis* seed dormancy and how to optimize propagation techniques to improve the germination rate and seedling survival rate.

## 6. Patents

We applied for a patent for the method of breaking dormancy and the rapid germination of *Parrotia subaequalis* seeds. Application (Patent) Number: ZL202310146507.7.

## Figures and Tables

**Table 1:** Physical characteristics of *P. subaequalis* seeds.

Length/mm		Short Diameter/mm		Seed Mass of a Thousand Seeds/g	
Mean ± Standard	Deviation Range	Mean ± Standard	Deviation Range	Mean ± Standard	Deviation Range
7.40 ± 0.46	5.70–8.30	3.17 ± 0.13	2.80–3.50	35.22 ± 0.24	34.80–35.40

**Table 2:** Effects of different concentrations of extracts on *B. rapa* var. *glabra a* germination.

Concentration	Germination Percentage, %	Germination Potential, %	Germination Index	Root Length/mm
Control	85.4 ± 1.2 ^a^	84.6 ± 4.6 ^a^	16.38 ± 3.2 ^a^	2.47 ± 0.7 ^a^
T1	77.4 ± 0.9 ^b^	77.3 ± 3.1 ^ab^	12.87 ± 2.0 ^ab^	2.05 ± 0.9 ^b^
T2	68.6 ± 1.8 ^c^	70.0 ± 4.0 ^b^	9.89 ± 5.4 ^ab^	1.12 ± 0.4 ^c^
T3	45.4 ± 4.1 ^d^	45.3 ± 14.1 ^c^	6.90 ± 1.4 ^b^	0.77 ± 0.3 ^d^
T4	21.4 ± 0.7 ^e^	21.3 ± 2.3 ^d^	6.68 ± 1.2 ^b^	0.44 ± 0.2 ^e^

The superscript letters in the table indicate significant differences among the values within the same column (*p* < 0.05).

**Table 3:** Germination of seeds treated with dry after-ripening.

Dry Storage After-Ripening Period/Days	Final Germination Percentage
0	17.3 ± 1.2 ^b^
30	22.7 ± 3.1 ^a^
60	23.3 ± 2.3 ^a^
90	25.3 ± 1.2 ^a^
120	21.3 ± 3.1 ^ab^

The superscript letters in the table indicate significant differences among the values within the same column (*p* < 0.05).

**Table 4:** Seed germination after cold and warm stratification treatment.

Stratification Temperature/°C	Stratification Duration/Days	Final Germination Percentage
0	0	17.3 ± 1.2 ^a^
4	30	18.0 ± 0 ^a^
	60	19.3 ± 4.3 ^a^
	90	20.0 ± 2.0 ^ab^
	120	23.7 ± 3.1 ^b^
15	30	20.7 ± 2.3 ^ab^
	60	21.3 ± 3.1 ^ab^
	90	23.3 ± 1.2 ^c^
	120	28.7 ± 3.1 ^d^

The superscript letters in the table indicate significant differences among the values within the same column (*p* < 0.05).

## Data Availability

The original contributions presented in the study are included in the article, further inquiries can be directed to the corresponding author.
